# *Candida auris* in the UK: Introduction, dissemination, and control

**DOI:** 10.1371/journal.ppat.1008563

**Published:** 2020-07-30

**Authors:** Andrew M. Borman, Elizabeth M. Johnson

**Affiliations:** UK National Mycology Reference Laboratory, National Infections Service, Public Health England, Science Quarter, Southmead Hospital, Bristol, United Kingdom and MRC Centre for Medical Mycology, University of Exeter, United Kingdom; McGill University, CANADA

*Candida auris* is an emerging yeast that has been reported as a cause of human infections in over 30 countries and 6 continents since its first description 10 years ago [1–12]. Capable of causing large nosocomial outbreaks, usually in high-dependency units [3–12], *C*. *auris* has the ability to colonize patients almost indefinitely and to contaminate hospital environments and medical equipment [6, 8]. Whole-genome sequence (WGS) analyses have revealed the existence of at least 4 phylogenetically separate clonal lineages of *C*. *auris*, each with firm associations with distinct geographic regions: clade I (Southern Asia), clade II (East Asia), clade III (South Africa), and clade IV (South America) [9]. WGS has revealed minimal diversity between isolates within each clade but vast sequence divergence between different clades, consistent with their almost simultaneous and very recent emergence as human colonizers/pathogens in multiple geographic areas [9]. Subsequent outbreaks reported in the United Kingdom, Central and Southern Europe, and North America have all been seeded by isolates that can be mapped genetically to 1 of these 4 clonal lineages [6–8, 10–13]. Interestingly, a potential fifth clade was recently described from a case of otitis in Iran [14], although to date, this has not been responsible for any nosocomial outbreaks.

## The introduction of *C*. *auris* into the UK and subsequent near-nationwide spread

**Fig 1:**
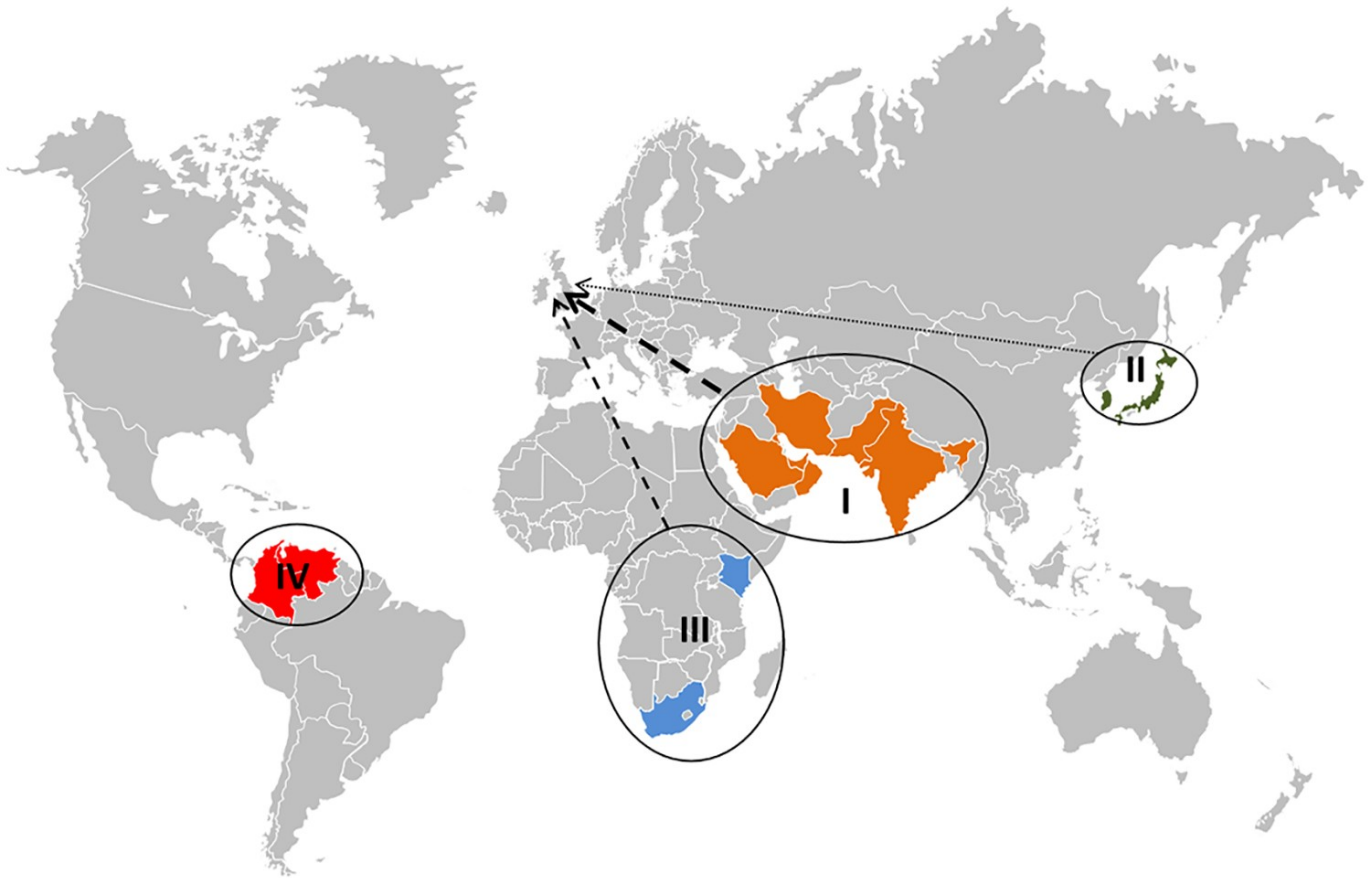
World map depicting the purported origins of the 4 major *Candida auris* clades; clade I (Southern Asia), clade II (East Asia), clade III (South Africa), and clade IV (South America). The dashed arrows represent clades that have been introduced into the UK, with line thickness proportional to the estimated numbers of separate introductions. (*World map by*: www.freeworldmaps.net https://www.freeworldmaps.net/powerpoint/).

Irrespective of whether the 4 major clonal lineages identified to date originated in South Asia, South Africa, South America, and East Asia or were transported there from elsewhere and found conditions favorable to their amplification, it is clear that all outbreaks and individual cases of *C*. *auris* infection worldwide can be traced back to isolates from those lineages [6–8, 10–13]. A retrospective analysis of historical isolates of unusual *Candida* species performed at the Public Health England (PHE) UK National Mycology Reference Laboratory (MRL) failed to find any evidence of *C*. *auris* in the UK prior to 2013. In 2013, the MRL received the first 3 isolates of *C*. *auris*, which originated from blood cultures from unrelated patients in 3 geographically separated healthcare centers [12]. A further isolate was referred to our laboratory in 2014, with 15 additional isolates (9 from sterile/deep sites) in 2015. By mid-2019, PHE had recorded approximately 270 cases of *C*. *auris* in England, including at least 35 cases of candidemia [15]. The majority of noninvasive cases involved patients colonized with *C*. *auris*, which were detected during enhanced screening of patients at 3 large hospitals in Southern England that experienced protracted outbreaks [6,10,13]. Genetic analyses have confirmed that 3 of the 4 clonal lineages (South Asia, South Africa, and East Asia) have been introduced multiple times into the UK [12] (Fig 1), with the widespread outbreaks fueled by isolates of the South Asian and South African lineages [6,10,13], and isolates from 2 different clades reported simultaneously in several centers [13,15]. Although all major outbreaks in the UK have, to date, been controlled, sporadic introductions still occur, mainly associated with patients who have direct travel links or healthcare stays in “endemic” areas (India, Pakistan, Oman, Kuwait, Qatar, Kenya). However, most of these recent introductions have not been associated with significant onward spread (< 5 cases; [15]), at least, in part, because of increased awareness, early recognition of cases, and effective isolation of affected patients and enhanced ongoing infection control measures. The vast majority of the approximately 270 isolates of *C*. *auris* detected in the UK have been recovered from patients in the South of England (metropolitan London and surrounding areas), with no cases reported from Northern Ireland or Scotland. We believe that this skewed pattern of introductions/detections probably reflects the facts that London Heathrow airport is Europe’s largest hub airport serving Asia, and the 3 hospitals affected by protracted outbreaks are large tertiary/quaternary centers in the vicinity of this major airport that provide extremely specialist services. As a result, *C*. *auris* cases in England predominantly appeared in specialist hospitals that clustered around the principal entry points of people traveling to and from endemic areas, which, to an extent, also reflects the geographical spread of immigrant communities.

## Identification issues in the UK healthcare system

Early reports concerning *C*. *auris* underscored the difficulties encountered in accurately identifying this emerging pathogen using conventional biochemical and phenotypic methods [3,4,7,16], with isolates frequently misidentified as a variety of unrelated yeast species. In 2017, a laboratory questionnaire circulated by the English surveillance program for antimicrobial utilization and resistance (ESPAUR) revealed that only 23 of the 58 English laboratories that responded were currently able to accurately identify *C*. *auris* on site [16]. National guidance for laboratory investigation of suspected isolates of *C*. *auris* was rapidly produced and disseminated widely [17], which encouraged laboratories without sufficient in-house expertise to submit all suspect isolates to the MRL or other reference laboratories for confirmation. Before the end of 2017, laboratories that employed matrix-assisted laser desorption/ionization-time of flight mass spectrometry (MALDI-TOF MS) for yeast identification using the Bruker platform were theoretically able to adequately identify this emerging pathogen. However, the Vitek MS platform was still unable to reliably identify *C*. *auris* isolates, and this capacity had only recently been introduced to Vitek 2 via a database update [7,16].

In response to the aforementioned developments, a panel of reference laboratory isolates of *C*. *auris* encompassing the 3 clonal lineages detected in the UK (plus control strains of other *Candida* species) was assembled by the MRL and distributed to 2 laboratories that employed both Vitek MS and Vitek 2 for yeast identification [16]. The results of independent testing in both laboratories confirmed that Vitek 2 with database v8.01 correctly identified isolates from all 3 *C*. *auris* clades [16]. From 2013 onwards, all suspected isolates of *C*. *auris* referred to the MRL were successfully identified by MALDI-TOF MS using the Bruker platform and an in-house extended database generated and curated by the MRL. In addition, the MRL developed a rapid molecular approach based on sequencing of 2 regions of the conserved rDNA gene cassette and phylogenetic analyses of resulting sequences that permitted the identification of the clonal lineage to which isolates belonged [12]. Finally, the MRL has recently investigated the utility of a novel chromogenic agar (CHROMagar Candida Plus) developed specifically to facilitate detection and identification of *C*. *auris*. Preliminary results indicate that this new medium performs well as a primary isolation medium and permits *C*. *auris* to be differentiated from over 50 other yeast species in *Candida* and related genera.

## UK Public Health responses to *C*. *auris*

Following the identification of 2 English hospitals with the first heathcare-associated outbreaks of *C*. *auris* in Europe [6,10], in 2016, a national incident management team (IMT) was convened by PHE in order to develop a consistent national response to this emerging nosocomial pathogen. The panel comprised representatives form a wide range of specialties, in order to provide guidance on the clinical management of affected patients, wider public health approaches, and infection prevention and control. The IMT included hospital microbiologists from the affected centers, infection prevention and infectious disease specialists, senior members of the MRL, public health experts, epidemiologists, and environmental microbiologists. Fortnightly teleconference meetings were held, some of which also included members of the Centers for Disease Control (CDC), Atlanta, and national guidance on all aspects of *C*. *auris* management was produced [17]. Members of the UK IMT also contributed to European guidance documents developed by the European Centre for Disease Prevention and Control (ECDC). The frequency of IMT meetings was gradually reduced over the next 24 months in response to increases in national knowledge and experience and the development of a wide range of mechanisms to monitor the evolution of local outbreaks and the national situation.

Additional national initiatives were also instigated. A multitiered system for reporting novel *C*. *auris* detections was developed, which included individual laboratories reporting directly into a national infectious diseases surveillance system, local health protection teams confronted by new incidents informing the IMT of new acquisitions, and the MRL feeding details of all isolates confirmed at the laboratory and especially those that represented new centers in real time directly to the IMT. In addition, national experts in healthcare-associated infection, environmental, biology and public health undertook site visits to hospitals affected by outbreaks, and guidance documents were updated regularly and expanded in line with increasing knowledge. Finally, several WGS initiatives were instituted, involving collections of isolates from all 3 English hospitals with extensive ongoing outbreaks. The results of WGS demonstrated that some apparent failures to control outbreak progression were in fact due to repeated independent novel introductions into a single center [10, 15]. Importantly, WGS also provided a detailed insight for the first time into a potential driver of nosocomial acquisition in one of the affected centers, by showing that reusable skin-surface axillary temperature probes harbored *C*. *auris* isolates that were indistinguishable from those found on some colonized patients [6]. Withdrawal of these probes halted the outbreak.

## How unusual is *C*. *auris*?

*C*. *auris* is the first human pathogenic fungus to be subject to international health alerts because of its propensity to colonize skin, persist in the hospital environment, cause nosocomial outbreaks, and cause severe disease. The ability of *C*. *auris* to colonize patients to extremely high levels [3–10] is certainly novel among known *Candida* spp. The preferential colonization of certain superficial body sites (axilla, groin) but not the intestinal tract fits well with the thermo- and halotolerance of the organism and its inability to prosper in anaerobic or acidic conditions [18] but again, is in contrast to most *Candida* infections that are acquired in hospital, which result from a patient’s own microbiome, often in the gastrointestinal tract. In addition, the finding that heavily colonized patients shed large quantities of *C*. *auris* (presumably via skin squames) into the environment and that the degree of shedding can be directly tied to the onward attack rate (i.e., the likelihood of that patient resulting in the colonization/infection of others on the same hospital unit) certainly sets *C*. *auris* aside from most other yeast species encountered in the clinical arena [13].

At first sight, the clonal nature of *C*. *auris* populations also appears novel, especially because the different clonal populations appear to possess different phenotypic and virulence traits and antifungal susceptibility profiles [19]. However, similarly distinct clades that had diverged distantly in the past have been described in another haploid human pathogen, *C*. *glabrata* [20]. However, the clonal lineages in *C*. *glabrata* have been extensively re-distributed worldwide and become intermingled, presumably as a result of global changes in travel and trade [20]. The same is rapidly becoming true with *C*. *auris*, with isolates from the 4 known clonal lineages being introduced into countries worldwide [6–13]. Thus, perhaps what is actually most novel with *C*. *auris* is that improved identification and strain-typing methodologies have allowed mycologists to track the global spread of *C*. *auris* in real time as it emerged as a human pathogen on the various continents. Future strain-typing approaches should be further facilitated by the recent development of a typing method based on short tandem repeat analyses [21]. It is certain that the geographically restricted nature of *C*. *auris* clades will rapidly become lost as isolates continue to be dispersed worldwide.

## What is the future for *C*. *auris* in the UK?

Recently, a point prevalence survey was conducted in England to attempt to ascertain population carriage of *C*. *auris*. No *C*. *auris* colonization was detected among approximately 1,000 patients who were screened at the time of admission to intensive care units (ICUs) at 8 English hospitals chosen on the basis of the ethnic populations that they served [15]. However, it is likely that this failure to detect *C*. *auris* in the prehospitalized population reflects the fact that the levels of carriage of this organism are below the limits of detection in patients who have not had protracted ICU stays and long courses of broad spectrum antibiotics. Since the first reports of *C*. *auris* outbreaks in India and South Africa, it has become widely accepted that this emerging pathogen has gained endemic status in many parts of South Asia and Sub-Saharan Africa [3,4]. Indeed, *C*. *auris* has become the third most common cause of candidemia in South Africa, affecting patients in >100 hospitals and causing >10% of all cases of invasive *Candida* disease [22]. As mentioned previously, although all major UK outbreaks have been controlled by aggressive screening, isolation, and infection control measures, there continue to be sporadic, novel introductions of *C*. *auris* into many English hospitals that can be directly traced to patients with travel from endemic areas. Of interest, one of our UK patients was airlifted back from Kenya following a road traffic accident and developed candidaemia due to the South African clade of *C*. *auris*; this did not lead to any further local cases, however, the patient still had colonization of the aural cavity 22 months later. We also see sporadic isolations referred from diabetic foot ulcers in London clinics suggesting some endemicity within the community, a phenomenon that has also been reported in India. This pattern of continued novel introductions into the UK, coupled with reports that affected patients frequently remain colonized with *C*. *auris* for long periods and perhaps even indefinitely [6,7,8,10], suggests that this emerging nosocomial pathogen is likely here to stay.

In the last month of 2019, a novel betacoronavirus (SARS-CoV-2) was reported as the etiological agent of a cluster of viral pneumonias (COVID-19) affecting the residents of Wuhan, China [23]. In the first 14 weeks of 2020 (at the time of writing), approximately 1.3 million cases of SARS-CoV-2 infection have been reported worldwide, with >70,000 deaths [24]. More than 180 countries and territories had been affected. Severe COVID-19 disease is associated with acute respiratory distress syndrome requiring ICU admission, mechanical ventilation or extracorporeal membrane oxygenation, protracted hospital stays, and significant mortality. Reports from several of the most seriously affected countries to date have indicated that cases of infection with SARS-CoV-2 have overwhelmed existing ICU capacity, with affected patients being managed in makeshift wards and hospitals where conventional infection control measures are challenging. Because previous studies have independently identified mechanical ventilation and stays in ventilator-assisted long-term acute hospitals as significant predictors of *C*. *auris* colonization/infection [8,25], the ongoing SARS-CoV-2 worldwide pandemic would appear to offer the perfect conditions for widespread novel outbreaks of *C*. *auris*.
